# Patient claims in prosthetic hip infections: a comparison of nationwide incidence in Sweden and patient insurance data

**DOI:** 10.1080/17453674.2018.1477708

**Published:** 2018-05-29

**Authors:** Piotr Kasina, Anders Enocson, Viktor Lindgren, Lasse J Lapidus

**Affiliations:** 1 Department of Clinical Science and Education, Södersjukhuset, Karolinska Institutet, Stockholm;;; 2 Department of Molecular Medicine and Surgery, Karolinska Institutet, Stockholm, Sweden

## Abstract

Background and purpose — Patients in Sweden are insured against avoidable patient injuries. Prosthetic joint infections (PJIs) resulting from intraoperative contamination are regarded as compensable by the Swedish public insurance system. According to the Patient Injury Act, healthcare personnel must inform patients about any injury resulting from treatment and the possibility of filing a claim. To analyze any under-reporting of claims and their outcome, we investigated patients’ claims of PJI in a nationwide setting

Patients and methods — The national cohort of PJI after primary total hip replacement, initially operated between 2005 and 2008, was established through cross-matching of registers and review of individual medical records. We analyzed 441 PJIs and the number of filed patients’ claims, with regards to incidence, outcome, and any national, sex-linked or socioeconomic differences.

Results — We identified 329/441 (75%) patients with PJIs as non-claimants. 96% of the filed claims were accepted. 64 (57%) of claimants sustained permanent disability. 2 factors were found to statistically significantly reduce the odds of filing claims: patient’s age above 73 years and fracture as indication for surgery. There were no significant national, sex-linked, or socioeconomic differences.

Interpretation — The incidence of patients’ claims of PJI is low but claims are usually accepted when filed. Healthcare personnel should increase their knowledge of the Patient Injury Act to inform patients about possibilities of eligible compensation.

Everyone treated in Sweden’s publicly financed healthcare is insured against injury resulting from avoidable patient injuries. Prosthetic joint infections (PJIs) are serious complications and may lead to severe consequences for those patients affected. PJIs after total hip replacement (THR) surgery, resulting from intraoperative contamination and not from hematogenous spread, are considered as compensable injuries by the Swedish no-fault insurance system.

During the last decade over 16,500 primary THRs were performed in Sweden each year (Karrholm et al. 2016). A study of primary THRs operated between 2005 and 2008 showed that the incidence of PJIs in Sweden, up to 2 years after surgery, is 0.9% (Lindgren et al. 2014).

The Swedish Patient Safety Act obligates healthcare personnel to inform patients about the possibility of filing a malpractice claim (SR-PSA 2017). Knowledge of the act varies among healthcare personnel (Espersson and Hellbacher 2016). Therefore there may be an under-reporting of patient injuries and consequently patients are not compensated to the extent intended by both the healthcare and legal system.

The aim of this study was to determine the proportion of filed claims and the outcome of these claims (accepted, rejected, and disability). We also analyzed any presence of national, socioeconomic, age, and sex-linked differences in patient claims.

## The patient insurance scheme in Sweden

Patients’ claims are handled by the national patient insurance company Landstingens Ömsesidiga Försäkringsbolag (LÖF), founded in 1975 and co-owned by the 21 Swedish county councils (public healthcare). LÖF handles all medical professions according to the Patient Injury Act (SR-PIA 2017) and regardless of the actual healthcare provider being a public, county council owned unit or a private division, delivering healthcare after procurement. The numbers of claims are increasing annually and in 2016 LÖF handled 16,000 claims with almost one-third of reimbursed claims being injuries related to orthopedic procedures (LÖF 2017). Moreover, THA is the procedure associated with the highest numbers of claims (Pukk-Härenstam et al. 2008). According to conditions of insurance, 6 types of injuries are covered, resulting from: treatment injury, technical damage, inferior diagnostics, infection, patient accidents in care, and medication-related injury. Patients report their injuries free of charge by completing a simple form, which must be filed within 3 years of injury detection but can under certain circumstances be accepted up to 10 years after treatment. LÖF then obtains full medical records before review of claims by specialists with expertise within the medical field concerned. In a case where the event was not avoidable and no causative relation or inferiority between given treatment and outcome is observed, LÖF can reject a claim. If the opposite is observed, LÖF compensates for the prolonged recovery time or awards payouts for sustained permanent disability, in accordance with Insurance Sweden consensus tables (IS 2014).

The economic compensation is non-tort, reimbursing income loss, unreimbursed medical costs and also includes the possibility to recompense for pain and suffering caused by the injury. It is also blame-free for practitioners and no records are shared with the regulatory authorities. Therefore it is neither punitive damage compensation nor a sanctioning tool of healthcare providers but supplements the extensive coverage offered by Swedish social and medical care systems.

## Patients and methods

### Data collection

This study is based on a previously described population (Lindgren et al. 2014) regarding the national incidence of PJI after primary THR in Sweden. All patients who had undergone a primary THR, between July 1, 2005 December and 31, 2008, were initially retrieved from the Swedish Hip Arthroplasty Register (SHAR), n = 49,219. Their Swedish personal identification number was used to match patients with the Swedish Prescribed Drug Register for continuous outpatient antibiotic medication for at least 4 weeks after surgery. The observation time was set to 2 years postoperatively to include only early and delayed PJIs (Zimmerli and Ochsner 2003). This protocol allowed for inclusion of the compensable PJIs, since intraoperative contamination occurs within the first 2 years. Simultaneously the late appearing PJIs caused by hematogenous spread were excluded. No additional selection was made to exclude any possible hematogenously spread infections within the first 2 postoperative years. Antibiotic treatments with indications other then PJI were excluded. A questionnaire for each of the identified infected 2,217 patients, with long postoperative antibiotic consumption, was sent to the operating units. This verified treatment for PJI and the case-specific diagnostic criteria of PJI, according to the definition by the Workgroup of the Musculoskeletal Infection Society (Parvizi et al. 2011). 99% of all questionnaires were returned and Lindgren et al. (2014) concluded a final number of 443 treated PJIs.

Consequently, we could identify 441 PJIs in LISA (the national agency Statistics Sweden’s longitudinal integration database for health insurance and labor market studies). This enabled matching on level of education as a socioeconomic factor. Finally, we compared all PJIs with LÖF’s database in November 2016 for patients’ claims and outcome of claims review. The timeframe was regarded as sufficient both for patients to file claims after delayed infections in case of complicated and prolonged PJI treatment and also for LÖF to review and conclude its decision.

### Study variables

For each patient, we recorded sex, age at primary THR operation, educational level, treating hospital, and the indication for surgery. SHAR divides these indications into 8 groups. Primary osteoarthritis and hip fracture are the 2 most common indications, with apparent different patient characteristics. Consequently we analyzed these 2 groups separately. The other 6 indications (inflammatory joint disease, sequel to pediatric disease, idiopathic femoral head necrosis, secondary osteoarthritis after trauma, malignancy, and other secondary osteoarthritis) each consist of fewer patients and are all mainly operated in elective settings. They were therefore merged together into 1 group, called “other”.

Highest achieved educational level was also grouped, from initial 7 levels (elementary school <9 years, elementary school >9 years, 3 years’ high school, postgraduate <3 years, postgraduate >3 years, doctoral education, and unknown) to 4 levels: elementary school, high school, postgraduate, and unknown.

To examine any national differences, each operating unit was classified according to its location by provision of care (the 21 Swedish counties) and separately by order: university hospital, referral county hospital, local hospital, and private.

Filed patient claims at LÖF were recorded and their decisions were grouped into 6 outcomes: rejected, prolonged recovery (< 3 months and >3 months), and permanent disability (1–15%, 16–30%, and >30%). 

### Statistics

The Mann–Whitney U test was used for the age variable in independent groups and Pearson’s chi-square test was used for nominal variables. Logistic regression analysis was performed on patients’ characteristics to evaluate factors associated with insurance claims. We used both a univariable and multivariable model. The former includes age, sex, diagnosis, and level of education, and the latter model consists of age and diagnosis. These 2 factors were verified as statistically significant in the univariable model and are simultaneously important in a clinical setting. Associations are presented as odds ratios (ORs) and risk ratios (RRs) with 95% confidence intervals (CIs). We used IBM SPSS Statistics software (ver. 23) (IBM Corp, Armonk, NY, USA).

### Ethics, funding, and potential conflicts of interest

The study was conducted in accordance with the Declaration of Helsinki and the protocol was approved by the Ethical Review Board in Gothenburg, Sweden (622-16).

We received support from LÖF, in the form of salary support for PK. LL has previously been involved in LÖF’s expert reviews but is not currently committed. There are no other conflicts of interest.

## Results

329 (75%) of the 441 patients with PJIs did not file a claim for injury with LÖF. Of those 112 that did, 108 (96%) were accepted as eligible for compensation (Table 1). Patients’ age above the median of 72 years (OR =0.4, CI 0.3–0.7, p = < 0.01) and fracture diagnosis (OR =0.4, CI 0.2–0.9, p = 0.02) were the only significant factors associated with not filing a claim for injury in a univariate logistic regression model (Table 2). There was also a tendency toward higher rates of claims among female patients. When adjusted for age, sex, diagnosis, and level of education in the later multivariate model, they remained significant, with similar OR, CI, and p-values.

**Table 1.** Baseline data for all patients included in relation to claim of injury at LÖF. Values are number (percentage) unless otherwise stated

	All	Claim made	Univariable		Multivariable		
	n	n (%)	ORa (95%CI)	p-value	ORa (95%CI)	RRb (95%CI)	p-value
Age							
< 73 years	224	74 (33)	1 (reference)		1 (reference)		
≥ 73 years	217	38 (18)	0.4 (0.3–0.7)	< 0.01	0.4 (0.3–0.7)	0.5 (0.4–0.8)	< 0.01
Sex							
male	219	48 (22)	1 (reference)				
female	222	64 (29)	1.4 (0.9–2.2)	0.1			
Diagnosis							
primary osteoarthritis	328	92 (28)	1 (reference)		1 (reference)		
hip fracture	67	9 (13)	0.4 (0.2–0.8)	0.02	0.4 (0.2–0.8)	0.5 (0.3–0.8)	0.02
other	46	11 (24)	0.8 (0.4–1.7)	0.6	0.7 (0.3–1.4)	0.8 (0.4–1.3)	0.3
Level of educationc							
elementary school	205	44 (22)	1 (reference)				
secondary school	167	48 (29)	1.5 (0.9–2.4)	0.1			
university	66	20 (30)	1.6 (0.9–3.0)	0.1			

**Table 2.** Logistic regression to evaluate factors associated with insurance claim

	All	Claim made	Univariable		Multivariable		
	n	n (%)	OR^a^ (95%CI)	p-value	OR^a^ (95%CI)	RR^b^ (95%CI)	p-value
Age							
< 73 years	224	74 (33)	1 (reference)		1 (reference)		
≥ 73 years	217	38 (18)	0.4 (0.3–0.7)	< 0.01	0.4 (0.3–0.7)	0.5 (0.4–0.8)	< 0.01
Sex							
male	219	48 (22)	1 (reference)				
female	222	64 (29)	1.4 (0.9–2.2)	0.1			
Diagnosis							
primary osteoarthritis	328	92 (28)	1 (reference)		1 (reference)		
hip fracture	67	9 (13)	0.4 (0.2–0.8)	0.02	0.4 (0.2–0.8)	0.5 (0.3–0.8)	0.02
other	46	11 (24)	0.8 (0.4–1.7)	0.6	0.7 (0.3–1.4)	0.8 (0.4–1.3)	0.3
Level of education^c^							
elementary school	205	44 (22)	1 (reference)				
secondary school	167	48 (29)	1.5 (0.9–2.4)	0.1			
university	66	20 (30)	1.6 (0.9–3.0)	0.1			

**^a^**OR = odds ratio.

**^b^**RR = risk ratio.

**^c^**3 missing values.

The variation in claims between counties ranged between 0% and 50%. Patients from smaller hospitals filed claims more often, with the highest rates from the private hospitals (34%) and the lowest from university hospitals (16%) (p = 0.06). We could not observe any statistically significant correlations between age of patients and claim rates from the different types of hospitals. Private hospitals operated only on patients with osteoarthritis (except 1 patient with femoral head necrosis).

44/112 (39%) claimants were compensated for prolonged recovery time and 64 (57%) for permanent disability, due to PJI (Figure). There was an equal distribution of age groups within the 3 largest decision groups: recovery <3 months, recovery >3 months, and disability 1–15%.

LÖF’s claims grouped by decisions (n) in the 112 claimed cases with distribution of age groups within.

## Discussion

Our main finding was the high incidence of non-claimants and that almost all filed claims were accepted for compensation by LÖF. This relation indicates on the one hand that those few patients who are claiming an injury from PJI are well informed about eligibility for compensation, but on the other hand it also suggests insufficiency of information from healthcare personnel to the large number of patients who do not make a claim. It is tempting to explain this by deficient knowledge of the Patient Injury Act among the personnel. An explanation may be a scarce awareness of legal obligations concerning patients’ information. Another is a possible incorrect impression of the system not being blame-free for practitioners, with a following unwillingness to report on colleagues if informing patients about LÖF. Third, in cases of fast and relatively complication-free recovery, healthcare personnel may not inform patients based on their own judgment, an assumption of certain cases not being severe enough to generate compensation. There may also be other reasons contributing to not filing a claim; several of these have been discussed in previous publications (Sager et al. 1990, Studdert et al. 2000, Bismark et al. 2006, Järvelin et al. 2012, Zengerink et al. 2016).

Although intraoperative contamination and hematogenously spread infections are not specified in our cohort, the probability of receiving compensation for PJI is high. We believe hematogenous spread was limited by the postoperative observation time and most PJIs are a result of IC (Zimmerli and Ochsner 2003, Azzam et al. 2010, Lee et al. 2010, Blomfeldt et al. 2015, Sendi et al. 2017). Additionally, 59% of claimants suffered a permanent disability, which also has a great impact on compensation.

Age above 73 years and fracture diagnosis were the 2 significant factors associated with lower rates of filed claims. Higher rates of non-claimants in the elderly correspond to previous findings (Sager et al. 1990, Studdert et al. 2000, Bismark et al. 2006, Järvelin et al. 2012, Zengerink et al. 2016). To our knowledge, specific claim rates of THR after fracture have not been studied previously. This finding suggests that healthcare personnel may be less prone to inform elderly patients, with possibly higher comorbidity, about the insurance.

Increased age and fracture diagnosis, possibly due to associated comorbidities, are associated with poorer outcome after treatment of PJI (Azzam et al. 2010, Blomfeldt et al. 2015). Our cohort included only 9 claimants who suffered a fracture and did not allow for any further analysis. Age was not observed to affect LÖF’s decisions in our study. Since the overall incidence of claims was low, it is not possible to draw any conclusions concerning outcome after PJI in this population. A possible explanation for our equal distribution of claim outcomes is that the more frail patients never filed a claim.

Our finding of a trend among women to be more prone to filing a claim is supported by the earlier studies of claims after THR (Järvelin et al. 2012, Zengerink et al. 2016) and claims in the general population (Bismark et al. 2006).

Another trend is the higher rate of claimants among patients operated in private hospitals. Their population of elective patients, commonly healthier and more aware of their rights and entitlements, may explain this. The private units may also have a better dialogue with their patients, which could also be a contributive factor.

Swedish compensation is substantially lower than seen in Anglo-American tort systems but simpler for the claimants and with higher overall appeal success rates (50 vs. 30%) (Kachalia et al. 2008, Pukk-Härenstam et al. 2008). This financial difference can be explained partly by the lack of a punitive component and partly by the existence of other forms of social insurance. The general support of the Swedish medical and social system may also diminish the economic importance of economic compensation. Additionally, elderly, injured, and often frail patients suffering from a PJI may refrain from filing claims due to perception of the process as difficult and long lasting. The fact of a free-of-charge, 2-page form and the relatively short time to decision (70% within 8 months) (Kachalia et al. 2008) is not obvious to the general population. Finally, patients may not realize the blame-free nature of the insurance. Some may refrain from claiming for their injury, as they are not willing to blame their doctor or department.

A limitation of our study is the inability to measure any rate of patient information about LÖF. We cannot conclude that non-claimants were not informed, nor can we assess the efficiency of delivery or quality of given information. Another limitation is the inability to measure any correlation between clinical outcome of PJI treatment and the likelihood of claiming injury. Additionally, we were not able to exclude hematogenously spread infections in our cohort beyond the limitation of the 2-year postoperative observation period. Therefore the presence of any hematogenously spread infections among non-claimants cannot be assessed. However, there were no such infections among the claimants and none of the 4 cases were rejected due to the route of contamination.

Nonetheless, we strongly suspect that most patients may not be aware that they have sustained an injury that is recognized by the healthcare system and possibly compensated by an insurance. It is therefore important to clearly inform patients suffering from PJI about their legal right to file a claim with LÖF and provide assistance when needed.

In summary our study shows that only every fourth PJI after THA is claimed as a patient injury. Simultaneously almost all claims were reimbursed by the Swedish national patient insurance, indicating that patients who file claims are informed about their rights. The low claim rate suggests insufficient patient information and is of concern from both a legal and a professional aspect.

All authors contributed to the conception and design of the study, analysis of data and its interpretation, as well as revision of the manuscript. PK and AE performed the statistical analysis. 

*Acta* thanks Pieter K Bos and Jutta Järvelin for help with peer review of this study.

## References

[CIT0001] Azzam K A, Seeley M, Ghanem E, Austin M S, Purtill J J, Parvizi J Irrigation and debridement in the management of prosthetic joint infection: traditional indications revisited. J Arthroplasty 2010; 25(7): 1022–7. 20378306 10.1016/j.arth.2010.01.104

[CIT0002] Bismark M, Brennan T A, Davis P B, Studdert D M Claiming behaviour in a no-fault system of medical injury: a descriptive analysis of claimants and non-claimants. Med J Aust 2006; 185(4): 203–7. 16922665 10.5694/j.1326-5377.2006.tb00532.x

[CIT0003] Blomfeldt R, Kasina P, Ottosson C, Enocson A, Lapidus L J Prosthetic joint infection following hip fracture and degenerative hip disorder: a cohort study of three thousand, eight hundred and seven consecutive hip arthroplasties with a minimum follow-up of five years. Int Orthop 2015; 39(11): 2091–6. 26381908 10.1007/s00264-015-2989-y

[CIT0004] Espersson C, Hellbacher U Patientskadelagen – en kommentar m. m. Vulkan, Riga 2016.

[CIT0005] Insurance Sweden IS. Medicinsk Invaliditet –skador 2013 Insurance Sweden 2014. Available from: http://www.svenskforsakring.se/globalassets/medicinska-tabellverk/medicinska-tabellverk/medicinsk_invaliditet_skador_rev_jan2014.pdf.

[CIT0006] Järvelin J, Häkkinen U, Rosenqvist G, Remes V Factors predisposing to claims and compensations for patient injuries following total hip and knee arthroplasty. Acta Orthop 2012; 83(2): 190–6. 22401679 10.3109/17453674.2012.672089 PMC3339536

[CIT0007] Kachalia A B, Mello M M, Brennan T A, Studdert D M Beyond negligence: avoidability and medical injury compensation. Soc Sci Med 2008; 66(2): 387–402. 17931762 10.1016/j.socscimed.2007.08.020

[CIT0008] Karrholm J, Lindahl H, Malchau H, Mohaddes M, Rogmark C, Rolfson O Swedish Hip Arthroplasty Register: Annual Report 2015 Gothenburg, Sweden 2016. Available from: https://registercentrum.blob.core.windows.net/shpr/r/Annual-Report-2015-H19dFINOW.pdf

[CIT0009] Lee J, Kang C I, Lee J H, Joung M, Moon S, Wi Y M, Chung D R, Ha C W, Song J H, Peck K R Risk factors for treatment failure in patients with prosthetic joint infections. J Hosp Infect 2010; 75(4): 273–6. 20635512

[CIT0010] Lindgren V, Gordon M, Wretenberg P, Kärrholm J, Garellick G Deep infection after total hip replacement: a method for national incidence surveillance. Infect Control Hosp Epidemiol 2014; 35(12): 1491–6. 25419771 10.1086/678600

[CIT0011] LÖF. Injury Report 2016. Anmälningar till LÖF 2016. Available from: http://lof.se/wp-content/uploads/Statistik-2016-Hela-Sverige.pdf. Landstingens Ömsesidiga Försäkringsbolag; 2017.

[CIT0012] Parvizi J, Zmistowski B, Berbari E F, Bauer T W, Springer B D, Della Valle C J, Garvin K L, Mont M A, Wongworawat M, Zalavras C G New definition for periprosthetic joint infection: from the Workgroup of the Musculoskeletal Infection Society. Clin Orthop Rel Res 2011; 469(11): 2992–4. 10.1007/s11999-011-2102-9 PMC3183178 21938532

[CIT0013] Pukk-Härenstam K, Ask J, Brommels M, Thor J, Penaloza R V, Gaffney F A Analysis of 23 364 patient-generated, physician reviewed malpractice claims from a non-tort, blamefree, national patient insurance system: lessons learned from Sweden. Qual Saf Health Care 2008; 17(4): 259-63. 10.1136/qshc.2007.022897 18678722

[CIT0014] Sager M, Voeks S, Drinka P, Langer E, Grimstad P Do the elderly sue physicians? Arch Intern Med 1990; 150(5): 1091–3. 2331189

[CIT0015] Sendi P, Lotscher PO, Kessler B, Graber P, Zimmerli W, Clauss M. Debridement and implant retention in the management of hip periprosthetic joint infection. Bone Joint J 2017; 99-B(3): 330–6. 28249972 10.1302/0301-620X.99B3.BJJ-2016-0609.R1

[CIT0016] SR-PIA. Sveriges Riksdag. Svensk författningssamling, Patientskadelag(1996:799) [The Swedish Parliament, Patient Injury Act], 2017:43. Available from: http://www.riksdagen.se/sv/dokument-lagar/dokument/svensk-forfattningssamling/patientskadelag-1996799_sfs-1996-799.

[CIT0017] SR-PSA. Sveriges Riksdag. Svensk författningssamling, Patientsäkerhetslag(2010:659). [The Swedish Parliament, Patient Safety Act], 2017:786. Available from: http://www.riksdagen.se/sv/dokument-lagar/dokument/svensk-forfattningssamling/patientsakerhetslag-2010659_sfs-2010-659.

[CIT0018] Studdert D M, Thomas E J, Burstin H R, Zbar B I, Orav E J, Brennan T A Negligent care and malpractice claiming behavior in Utah and Colorado. Med Care 2000; 38(3): 250–60. 10718350 10.1097/00005650-200003000-00002

[CIT0019] Zengerink I, Reijman M, Mathijssen N M, Eikens-Jansen M P, Bos P K Hip arthroplasty malpractice claims in the Netherlands: closed claim study 2000–2012. J Arthroplasty 2016; 31(9): 1890–3. 27062353 10.1016/j.arth.2016.02.055

[CIT0020] Zimmerli W, Oshsner P E Management of infection associated with prosthetic joints. Infection 2003; 31(2): 99–108. 12682815 10.1007/s15010-002-3079-9

